# Mr. Christie, on Vaccination

**Published:** 1807-06

**Authors:** James M'Grigor

**Affiliations:** Portsmouth


### *To the Editors of the Medical and Physical Journal.*

GENTLEMEN,

IF you have not entirely shut the door against further accounts of vaccination, the following may be acceptable to your readers; it is a statement by my friend, Mr. Christie, Superintendant General of Hospitals in Ceylon, which I have just received from him, and which he published in the Government Gazette.

I am, &c.

*Portsmouth, April* 20, 1807. JAMES M'GRIGOR.

---

THE number of inoculations in the vicinity of Columbo, has been greater in June than for many months past, in consequence of the appearance of small-pox, which broke out in the Bazar, and several other parts of this district in May last. I have not been able very distinctly to trace its origin, but as we have enjoyed an exemption from its influence here for nearly three years, and as it was imported from the coast to Aripo in February last, it seems most probable that the infection was brought from thence on the breaking up of the fishery in the end of April, more particularly as it is ascertained that patients were landed from Aripo with small-pox, both at Calpentyn and Chilow.

Although the disease appeared in several parts of this district at the same time, I am happy to say, that by means means of the precautions which have been taken, its propagation to any considerable extent has been prevented. As soon as the existence of small-pox in any village or street was ascertained, Vaccinators were immediately sent to the spot, to urge the people in the neighbourhood to submit to immediate inoculation; and when it was practicable, the patients infected with small-pox were removed to a secluded situation, near the Leper's Hospital, where every aid was afforded them.

I am happy to say, that for a great length of time there has been almost no prejudice against vaccination in this island, and that all the more intelligent inhabitants have the fullest confidence in it, as a certain preventive against small-pox; but as some futile attempts have lately been made in Europe, to shake the confidence of the public in the efficacy of this inestimable discovery, particularly by inculcating an idea that the constitution is not permanently shielded by its influence, but that, at the end of three years, a fresh inoculation may perhaps be necessary, it must prove satisfactory and I hope convincing to mention, that amongst the many thousands who were inoculated in 1802, and the succeeding years, there is not a suspicion that any one of them has been affected with small-pox, though many of them have been exposed in various ways to the most virulent contagion.

The existence of small-pox at the present period, has enabled us to put some of our patients to the test after the expiration of nearly four years from the time of their vaccination. Mr. Morton accordingly, in May last, when acting as superintendant of vaccination in this district, inoculated with active variolous virus two patients, Katto, a slave girl, and Johannes Fernando, a Cingalese, who had passed through the cow-pox in Sept. 1802; and many others have, by my directions, been exposed to the infection, without any effect but slight local inflammation on the inoculated part.

I have also, with the same event, inoculated with and exposed to small-pox, several of the patients vaccinated last month, particularly Carolus Rhode and Jonan Tedoe, residing at Moolwal, with a view to prove, that our infection, though it has passed on this island alone through a regular succession of at least two hundred patients, without any renewal from the cow, still possesses, unimpaired, its genuine qualities and preventive virtues.

The following instance exemplifies in so striking a manner these facts, that I cannot resist the pleasure of relating it.—

it.—On the morning of the 11th instant, when visiting with Dr. High, staff surgeon, the small-pox patients near the Lepers Hospital, I was informed that a boy in one of the neighbouring houses was affected with small-pox, which surprised me much, as I had been informed that, agreeably to my instructions, every person in the village, who had not had small-pox, had been vaccinated. We immediately repaired to the house, where we found a boy, named Gorkanize Savery, labouring under small-pox, on the fourth day of the eruption. On inquiry, I found he had not been inoculated, because he and his friends said, that four years before he had passed through an eruptive disease, believed to have been the small-pox. The family consisted of seven other persons, three of whom were aged, and had passed through the small-pox; two had been vaccinated in 1802, and the remaining two in May, 1806. The whole of them continued, by my direction, to live in the house with and attend on the boy with small-pox, who is now recovered, without having communicated the disease to any of them.

In a former letter I took occasion to mention, that, agreeably to the most certain information, at least one-third of all the inhabitants of this island, who were affected with natural small-pox, died. The proportion of deaths on the present occasion, justifies that calculation, as out of twenty patients who have been removed to the Hospital, and regularly treated, seven have died; and I have reason to believe, that the mortality amongst the patients, who remained in their own houses, has been still greater.

The number of vaccinated patients, reported to me since the introduction of cow-pox in Ceylon in August, 1802, amounted on the 30th of June, 1806, to 47,523; and if, agreeably to a moderate calculation which I formerly made, not more than one-half of the inhabitants of this island escaped natural small-pox, and of the half that had it, one-third died, we may, without over-rating the benefits of vaccination, fairly estimate, that of the 47,523 patients who have been inoculated in the island, one-sixth of the whole, or 7920, would have otherwise died of the small-pox, which, previous to the introduction of vaccination, was almost every year epidemic at Columbo, and many other parts of the island.

I shall conclude with mentioning one fact, which may prove interesting to your medical readers, and, as far as I know, is new in the history of vaccination. It is, that Peter Baro, a boy of European parents, who has long been confined with confirmed leprosy, and whose features are much much disfigured with that disease, was vaccinated on the 4th of June, 1806, and passed through the disease regularly.

It has with justice I think been supposed, that psora and other diseases of the skin diminished the susceptibility of cow-pox; and yet we find, in the present instance, a most inveterate disease did not prevent its taking effect, which ought to encourage us, in the event of the prevalence of small-pox, not to be deterred by the presence of other diseases, from giving vaccination a trial; for, had it not been practiced with this boy, there is reason to apprehend that he might have fallen a martyr to small-pox, which prevailed in his vicinity.

ABSTRACT of the Returns of Patients Inoculated for the Vaccine Disease in different Districts of Ceylon, during the month of June, 1806.

| DISTRICT. | Numbers Inoculated. | Discharged with Certificates. | Failed. | Remaining. |
|---|---|---|---|---|
| Colombo | 1018 | 679 | 230 | 109 |
| Caltura | 111 | 108 | | 3 |
| Galle | 248 | 88 | 1 | 159 |
| Matura | 213 | 122 | 27 | 64 |
| Tangalle | 200 | 143 | 10 | 47 |
| Hambangtotte | 60 | 26 | 4 | 30 |
| Batticaloa | 57 | 30 | 12 | 15 |
| Trincomallie | 41 | 21 | 6 | 14 |
| Mullativo | 36 | 25 | | 11 |
| Jaffna | 276 | 214 | | 62 |
| Manar | 117 | 65 | 14 | 38 |
| Calpetyn | 24 | 23 | 1 | |
| Chilou | 49 | 11 | 31 | 7 |
| Negumbo | 40 | 26 | 4 | 10 |
| Total | 2490 | 1581 | 340 | 569 |

Note, The proportion of failures is greater than usual in the Colombo district, in consequence of several people having been inoculated for the sake of security, who were uncertain as to their having had small-pox.

I am, &c.

T. CHRISTIE, Med. Sup. Gen.

*Colombo, July* 22, 1806.

